# Determination of the discriminant score of intestinal microbiota as a biomarker of disease activity in patients with ulcerative colitis

**DOI:** 10.1186/1471-230X-14-49

**Published:** 2014-03-19

**Authors:** Katsuyuki Fukuda, Yoshiyuki Fujita

**Affiliations:** 1 Department of Gastroenterology, St Luke’s International Hospital 9-1, Akashi Tokyo, cho, Chuo-ku 104-0044, Japan

**Keywords:** Intestinal microbiota, Ulcerative colitis, Terminal restriction fragment length polymorphism, Discriminant score, Operational-taxonomic-unit, Canonical discriminant function coefficient

## Abstract

**Background:**

In recent years, the gut microbiota has been found to provide an important link to the development of inflammatory bowel diseases (IBD) like ulcerative colitis (UC). Accordingly, inter-individual variation in the gut microbial community may be linked to inter-individual variation in the risk of IBD or other diseases. Further, the Terminal Restriction Fragment Length Polymorphism (T-RFLP) is a molecular biology technique for profiling bacterial species in faecal samples. This study was to evaluate a biomarker based on intestinal microbiota.

**Methods:**

The study subjects were 69 patients with UC together with 80 relatives as controls. Twenty-three patients had active UC (group I) and 46 had quiescent UC (group II). The later included 17 patients with mild inflammation in the large intestine (group IIa), 29 without inflammation (group IIb). The patients’ relatives were consanguineous (group III, n = 47), and non-consanguineous (group IV, n = 33). Faecal samples were obtained from all subjects for the investigation of intestinal microbiota by applying the T-RFLP method. The Discriminant analysis of operational-taxonomic-unit (OTU) on T-RFLP fingerprints was performed. The Canonical Discriminant Function Coefficient (Df) for each OTU was calculated. The individual OTUs were multiplied by the Df value, and the sum was termed the Discriminant Score (Ds).

**Results:**

The Ds decreased thus: group I > group IIa > group IIb > group III > group IV. Significant difference was calculated for group I vs group IV (P < 0.01), group I vs group IIb (P < 0.05), group I vs group III (P < 0.01), group IIa vs IV (P < 0.01), group IIb vs group IV (P < 0.01), group III vs group IV (P < 0.01), indicating a strong association between gut microbial species and the development of UC.

**Conclusions:**

In this study, the Ds related to UC, or otherwise absence of UC in the five groups. Potentially, Ds may become a clinically relevant biomarker of disease activity in UC. To our knowledge, this is the first application of the Ds to the study of microbiota in UC patients, consanguineous and non-consanguineous relatives.

**Trial registration:**

Clinical trial No: UMIN 000004123.

## Background

Inflammatory bowel disease (IBD) is a chronic relapsing-remitting intestinal immune disorder that afflicts millions of individuals throughout the world with debilitating symptoms like diarrhea, rectal bleeding, and weight loss, which impair function and quality of life [1]. IBD has two major phenotypes, ulcerative colitis (UC) and Crohn’s disease (CD). Although UC is primarily confined to the colon and the rectum, CD may affect any part of the gut from the mouth to the perianal region, and up to 65% of CD patients may have the disease affecting the small intestine. The aetiology of IBD and factors, which provoke flare-ups are not understood well at present and this might be one factor for treatment failure and adverse side effects of currently available medications.

However, the human intestine is host to thousands of bacterial species, collectively referred to as the intestinal microbiota (2). While it is understood that the presence of this microbiota is essential for the human health, this relationship may become unbalanced, resulting in diseases like UC and CD [2,3]. Thus, both clinical and experimental evidence suggest that IBD fare-ups are triggered by a combined loss of the so-called intestinal barrier function and a dysregulated immune response to the intestinal microbiota [2-12]. Accordingly, in animal models of IBD, experimental colitis is reported not to occur under bacteria-free condition [6,13,14]. This seems paradoxical as the human intestinal bacteria are known to metabolize compounds that might be essential for human nutrition [15,16]. Additionally, probiotics, which change the composition of intestinal microbiota have shown efficacy in patients with IBD [3], [17-22], suggesting that among the vast species of bacteria which are found in the human intestine, there are both pro-inflammatory [23,24], as well as protective strains [17].

Although, the evidence for the role of intestinal microbiota in the flare-up as well as in the mitigation of intestinal inflammation is strong, there is also evidence that patients with IBD bear genetic susceptibility factors [25,26], familial clustering and a high degree of IBD concordance [25-28]. Accordingly, there are families that have multiple relatives with CD or UC, which provides evidence for genetic factors in the aetiology of IBD [27].

Given that intestinal microbiota in patients with IBD may bear features different from those in healthy individuals, characterization of these bacterial communities should be a major focus of the studies aimed at understanding of IBD aetiology. Based on this perception, gut bacterial community fingerprinting technique like the terminal restriction fragment polymorphism (T-RFLP) analysis can be applied to determine inter-individual differences in the gut microbiota [29-31]. Data from T-RFLP investigations should provide clinically relevant information on the compositional differences of gut bacterial communities [30-32]. This article focuses on UC.

We thought that potentially, the application of T-RFLP to the bacteria in faecal samples from patients with UC may provide a biomarker to trace UC in community based studies. Ideally, we hoped that such biomarker should be related to UC severity and the likelihood of a flare-up. Further, the application of T-RFLP to interpret intestinal bacterial flora of UC patients and the patients’ relatives, which potentially could reveal the development of UC within a consanguineous generation (related by blood) has not been attempted in the past. Likewise, cluster analysis applied in T-RFLP studies is a relative evaluation, yielding equivocal data [32]. In line with this assertion, cluster analyses in the present study also provided uncertain outcome. Instead, we applied quantitative Discriminant analyses of intestinal microbiota of UC patients and the patients’ relatives. This approach provided clinically relevant data.

## Methods

### Subjects

Sixty-nine patients with UC, 41 female and 28 male, median age 46.5 years, range 13 to 79 years were included in this study. Additionally, an 80 relatives of the same patients, 40 female and 40 male, median age 45.2 years, range 2 to 78 years were included to broaden the scope of the study. The 69 UC patients could be divided into group I (n = 23) with active UC, clinical activity index (CAI) > 3 according to Lichtiger [32], and group II (n = 46) with UC in quiescent phase (CAI ≤ 3). Patients with quiescent UC could be divided further into 2 groups based on colonoscopic findings. Seventeen had mild inflammation in the large intestine (group IIa), and 29 had no obvious inflammation in the large intestine (group IIb). Likewise, the patients’ relatives could be divided into two subgroups. Patients’ akin (consanguineous relatives), like father, mother, a child, brother, or sister (group III, n = 47), and without blood relationship (non-consanguineous relatives), like husband, wife, father-in-law, mother-in-law (group IV, n = 33). Faecal samples were obtained from all subjects (Table 1) for the investigation of intestinal microbiota by applying the T-RFLP method (described below). All subjects were managed at the Division of Gastroenterology, St Luke’s International Hospital, Tokyo. This investigation was registered with the UMIN, No. 000004123 http://www.umin.ac.jp/.

**Table 1:** Demographic variables of the included subjects

**Subjects**	**Number of patient**	**Median age (yr)**
Group I: patients with active ulcerative colitis (UC)	23 (8 male, 15 female)	42.4
Group IIa: patients with quiescent UC with mild intestinal inflammation	17 (6 male, 11 female)	42.3
Group IIb: patients with quiescent UC	29 (14 male, 15 female)	52.3
Group III: patients’ consanguineous relatives	47 (23 male, 24 female)	40.1
Group IV: patients’ non-consanguineous relatives	33 (17 male, 16 female)	52.5

### Terminal restriction fragment length polymorphism (T-RFLP) procedures

T-RFLP is a molecular biology technique developed for profiling bacterial communities based on the position of a restriction site closest to a labeled end of an amplified gene [29]. T-RFLP involves sequentially breaking down a mixture of polymerase chain reaction (PCR) amplified variants of a single gene by using one or more restriction enzymes and detecting the size of each individual terminal fragments with the aid of a DNA-sequencer. Essentially, T-RFLP can be applied to generate a fingerprint of an unknown bacterial community and trace it through a family or population. Faecal samples were suspended in a solution containing 100 mM Tris–HCl, pH 9.0, 40 mM Tris-EDTA, pH 8.0, and 4 M guanidine thiocyanate, and kept at −20°C until DNA extraction. Aliquots of 0.8 ml faecal suspensions were homogenized with zirconia beads in a 2.0 ml screw cap tube by FastPrep FP120A Instrument (MP Biomedicals, Irvine, CA) and placed on ice. After centrifugation at 5000xg for 1 min, the supernatant was transferred to the automated nucleic acid isolation system 12GC, and DNA extraction was done by using the Magtration MagaZorb DNA Common Kit 200 N (Precision System Science, Chiba, Japan).

### PCR amplification for T-RFLP analysis

The 16S rDNA was amplified from human faecal bacterial DNA by using the fluorescently labeled 516f (5′-(6-FAM)-TGCCAGCAGCCGCGGTA-3′), and 1492r (5′-GGTTACCTTGTTACGACTT-3′) primers. For this, the Hot-starTaq DNA polymerase by Gene Amp PCR system 9600 (Applied Biosystems) was used [33]. The amplification program was as follows: preheating at 95°C for 15 min, 30 cycles of denaturation at 95°C for 30s, annealing at 50°C for 30s, extension at 72°C for 1 min, and finally, a terminal extension at 72°C for 10 min. The amplified DNA was pulyfied by a MultiScreen PCR96 Filter Plate (Millipore) and was verified by electrophoresis. The restriction enzymes were selected according to Nagashima et al. [34]. In brief, the PCR product was purified, and digested with BslI (New England BioLabs, Ipswich, USA). The resultant DNA fragments, viz., fluorecent labeled terminal restriction fragments (T-RFs), were analyzed by ABI PRISM 3130xl genetic analyzer, and its length and peak area were determined by using the genotype software GeneMapper (Applied Biosystems). The T-RFs were divided into 29 operational taxonomic units (OTUs). The OTUs were quantified as the percentage of individual OTU per total OTU areas, expressed as the percentage of area under the curve (%AUC). The bacteria were predicted for each classification unit and the corresponding OTU was identified according to reference Human Faecal Microbiota (T-RFLP profiling, http://www.tecsrg-lab.jp/).

### Ethical considerations

Before contacting the study subjects, our protocol was reviewed and approved by the Screening Committee of St Luke’s International Hospital (the study site). Likewise, informed consent was obtained from all patients and the patients’ relatives by using the consent explanatory note document, which is recognized by the Screening Committee of St Luke’s International Hospital. Subjects provided informed consent after being informed of the purpose of the study, and the nature of the procedures involved. In under age cases, consent from one of the patient’s parents was sought. Additionally, adherence was made to the Principle of Good Clinical Practice and the Declaration of Helsinki at all times.

### Statistics

When appropriate, numerical data are presented as the mean ± SD values. The Discriminant Score (Ds) was calculated according to the following mathematical equation, $\mathrm{Ds}=d+\sum_{j=1}^{m}\left(\mathrm{dj}\times \mathrm{\alpha j}\right)$ where m = number of OTUs, αj = value of each OTU, dj = Df value of each OTU, D = a constant and j is a variable. The Discriminant analysis was performed by using the software SPSS (IBM Statistics 20.0). The t-statistic was applied to determine significance levels for the male and female ratio and the age difference between groups I to IV. Bacterial communities in faecal samples from BslI-digested T-RF patterns in groups I to IV were processed by One Way ANOVA.

## Results

### Limitations of cluster analyses

**Figure 1:**
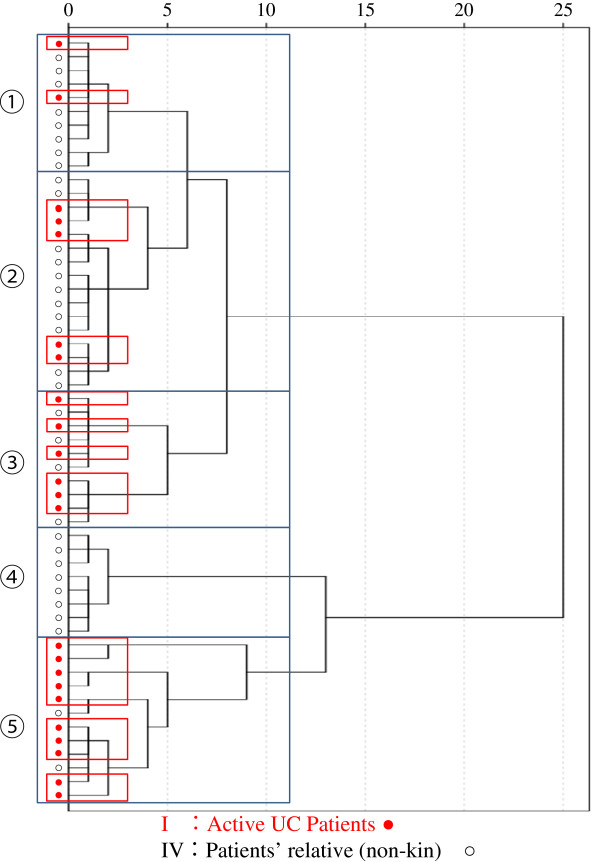
**Dendrograms based on ward linkage rescaled distance cluster combine.** The difference of intestinal flora was thought to be greatest between active phase patients (group I) and non-consanguineous families (group IV). However, as seen, cluster analysis between group I and group IV by using all OTU values did not produce visible separation between group I and IV. This was considered to be a serious limitation of cluster analysis.

**Figure 2:**
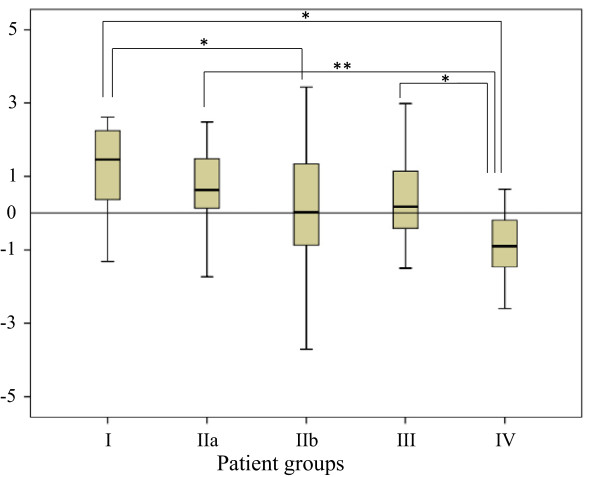
**The Discriminant Score (Ds) for group I to group IV was calculated by using all OTU values.** The Ds value was seen to decrease in the following order group I > group IIa > group IIb > group III > group IV, but the variation was large and the tendency was still uncertain. Group comparison was made by using One Way ANOVA, group I vs group IV (P < 0.01); group I vs group IIb (P < 0.05); group IIa vs group IV (P < 0.01); group III vs group IV (P < 0.05).

Hitherto, cluster analysis has routinely been applied to T-RFLP data [29-32], shown in Figure 1. Therefore, in this study, we initially applied cluster analysis to our data. Although practical on data from two groups, but cluster analysis appeared to be complicated and the outcome unreliable when data from five groups were to be processed. It was thought that the difference of intestinal microbiota between patients with active UC (group I) and non-consanguineous relatives (group IV) might be greater than between any other two groups. However, the cluster separation between groups I and IV was not apparent at all (Figure 2). We then thought that it might be realistic to apply the Discriminant analysis, which factors a mathematical model described in the statistics section.

### The outcomes of discriminant analysis

In this study, the Discriminant analysis was applied to the data as quantitative approach based on the mathematical model mentioned above. The Canonical Discriminant Function Coefficient (Df) for each OTU value and a constant (see the equation in the statistics section) were calculated by using group I and group IV, in whom the intestinal microbiota difference was thought to be greatest among the four groups of subject in this study. Then the individual OTU values were multiplied by the aforementioned Df, and the total sum including a constant was termed the Discriminant Score (Ds) of an individual case. The Ds values for all cases were computed. The results were lucid and meaningful. The Ds values decreased in the following order: group I > group IIa > group IIb > group III > group IV. Significant differences were calculated by One Way ANOVA for group I vs group IV (P < 0.01), group I vs group IIb (P < 0.05) group IIa vs group IV (P < 0.01) and group III vs group IV (P < 0.05). The outcomes are presented in Tables 2 and Figure 2.

**Table 2:** **The Discriminant Score (Ds) from all OTU values (see Figure**2**)**

**Subjects**	**Ds value (mean ± SD)**
Group I: Patients with active ulcerative colitis (UC)	1.24 ± 1.17
Group IIa: Patients with quiescent UC with mild intestinal inflammation	0.78 ± 1.76
Group IIb: Patients with quiescent UC (without inflammation)	-0.10 ± 2.43
Group III: Patients’ consanguineous relatives	0.25 ± 1.39
Group IV: Patients’ non- consanguineous relatives	-0.87 ± 0.87

**Figure 3:**
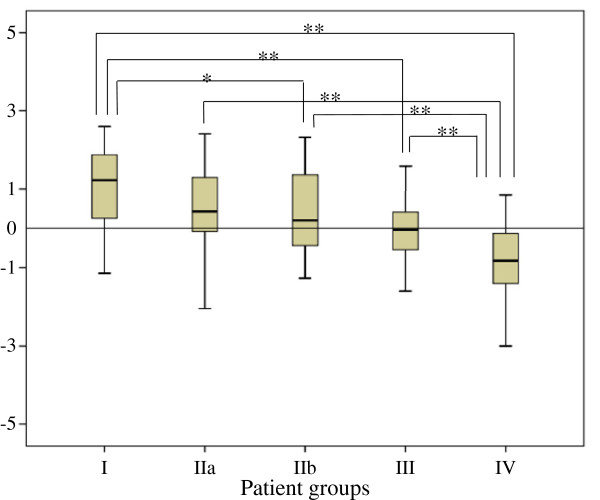
**The Discriminant Score (Ds) of each group, from group I to group IV was calculated by using select OTU.** As shown, Ds value decreased in the order: group I > group IIa > group IIb > group III > group IV, and the tendency became certain. Significant difference was calculated for group I vs. group IV (P < 0.01), group I vs. group IIb (P < 0.05), group I vs. group III (P < 0.01), group IIa vs. IV (P < 0.01), and group IIb vs. group IV (P < 0.01), group III vs. group IV (P < 0.01). See text for details.

Further, as certain strains of bacteria are thought to be associated with the development of UC, while others not so, this should show up in the OTU value in T-RFLP analysis. The analysis was set to factor this assumption, and the OTUs with 0 value ≥95% of all cases together with the OTUs of structure matrix value ≤0.01 according to the Discriminant analysis were excluded, and the Discriminant analysis was done (Figure 3). Again the Ds value decreased in the order: group I > group IIa > group IIb > group III > group IV. The numerical values of Ds were: 1.04 ± 1.15 for group I, 0.53 ± 1.40 for group IIa, 0.32 ± 1.00 for group IIb, 0.09 ± 0.84 for group III and −0.73 ± 0.88 for group IV. Significant difference was calculated for group I vs. group IV (P < 0.01), group I vs. group IIb (P < 0.05), group I vs. group III (P < 0.01), group IIa vs. IV (P < 0.01), and group IIb vs. group IV (P < 0.01), group III vs. group IV (P < 0.01). The data are presented in Table 3 and Figure 3. Clearly, the Ds value reflected UC disease activity (or lack of it) in the five groups of this study. Further, as the Ds value comes from the analyses on the intestinal microbiota and factors the OTU value, this parameter appears to be a clinically relevant biomarker of UC disease activity. Potentially, this means that analysis of bacterial flora by applying the mathematical model used in this study should become a routine practice. To our knowledge, this is the first report on the application of the Ds to the study of intestinal bacteria in faecal samples from UC patients’ consanguineous and non-consanguineous relatives.

**Table 3:** **The Discriminant Score (Ds) from selected OTUs related to the data presented in Figure**3

**Subjects**	**Ds value (mean ± SD)**
Group I	1.09 ± 1.13
Group IIa	0.69 ± 1.59
Group IIb	0.27 ± 1.05
Group III	0.03 ± 0.81
Group IV	-0.76 ± 0.90

## Discussion

The knowledge that the intestinal microbiota in patients with UC is a major factor in the aetiology of this condition is supported by animal models of colitis, reporting that the development of UC-like colitis did not occur under bacterial free conditions [14,15], but in the same setting, colitis occurred following introduction of gut bacteria from a UC patient [14]. In contrast, faecal bacteria from healthy donors are expected to have therapeutic effect in patients with IBD [35]. With this background in mind, we became interested to evaluate a biomarker based on the gut microbiota, being closely related to UC activity and potentially could indicate a UC relapse. The findings of this study might be summarized as follows. Our study subjects were divided into five groups and included patients with UC in active phase, in quiescent phase (with and without mild mucosal inflammation), UC patients’ consanguineous and non-consanguineous relatives. The latter two groups served as non-IBD controls with and without having blood relationship with the UC groups. In processing of the T-RFLP data, cluster analysis [34,36,37], which hitherto studies have frequently applied did not seem to work when applied to five groups in this study. Then we thought that it might be logical to apply the Discriminant analysis, together with a mathematical model (shown in the statistics section). Among the five groups, the Ds value was positive and greatest in group I with active UC, smallest and negative in group IV who had neither any evidence of IBD nor blood relationship with the UC patients. This was quite interesting as only in this group, the magnitude of the Ds value was negative. Therefore, the interpretation of the Ds values could mean that patients with UC and their non-IBD relatives share intestinal microbiota, which may not be present in non-IBD/non-consanguineous relatives. As the Ds value comes from the analyses on the intestinal microbiota, and factors the OTU value, the Ds appears to be a clinically relevant biomarker of UC disease activity. Potentially, this means that analysis of bacterial flora by applying the mathematical model used in this study should become a routine practice. Additionally, it should be appropriate to mention here that current treatment regimens for UC, which include corticosteroids, thiopurines (6-mercaptopurine, and azathioprine) or biologics such as infliximab that block the activity of the inflammatory cytokine tumour necrosis factor (TNF)-α have shown efficacy in this clinical setting, but are without effect on the underlying basis of the disease like dysbacteriosis.

Progress in understanding factors, which are closely associated with the expression and the exacerbation of UC should lead to better management of this disorder. Intestinal bacterial flora fingerprinting techniques like TRFLP analysis [29,30], potentially offer a rapid view of inter-individual differences in gut bacterial flora, and whether or not such differences define UC profile. When comparing the T-RFLP data obtained from different populations, variation can be found in the number and size of peaks and can be evaluated by selecting features like richness and evenness. The technique is designed to provide quantitative information on the compositional differences of intestinal bacteria with the potential to serve as a biomarker in population based studies. However, to be relevant as a biomarker, T-RFLP data need to be highly reproducible and reflect the composition of intestinal microbiota. In addition to the limitations of cluster analysis already mentioned, methodological approaches like sampling technique and DNA extraction, have the potential to influence the T-RFLP fingerprint of microbial communities [38]. Therefore, obtaining microbial genomic DNA that accurately represents the gut microbial community is essential [30,34]. When extracting genomic DNA from a complex matrix such as faeces, not only is extraction efficiency of genomic DNA from a wide variety of bacteria an essential consideration but removal of contaminants that co-elute with the DNA may interfere with the subsequent molecular analyses is necessary as well. With these limitations in mind, we applied the DNA extraction method described by Nagashima et al., which provides reproducible data [34].

In this study, our major endeavour was to evaluate a simple and reliable model for thorough investigation of the role of intestinal bacterial in the aetiology of UC [39,40]. All specimens needed could be extracted from faecal samples. However, given the fact that there are strain of bacteria, which are part of the aetiology of IBD, while other strains are protective [35,41], a comparison of the Ds values from the UC patients and non-IBD relatives in this study did not lead to the identification of a specific sequence or group of sequences exclusively harbored by UC patients. Therefore, it was not possible to relate certain bacterial species to the presence or absence of UC. The most obvious difference in the mucosa-associated flora from the UC and non-IBD patients was the absolute value of Ds. The Ds should be appropriate for assessing the clinical state and treatment policy in patients with UC. It was thought that in patients with a definitive diagnosis of UC, a low Ds value could indicate stable remission and vice versa. We also noticed that a high OTU value was related to the severity of active UC.

## Conclusions

Intestinal microbiota fingerprinting techniques, like the T-RFLP analysis, potentially offer a rapid overview of inter-individual differences in gut bacterial flora. Analyses of intestinal microflora of UC patients and their relatives based on T-RFLP, and the determination of the Ds values by using the selected OTUs indicated that the risk becomes high as the Ds value increases. This method should be valuable as a quantitative assessment of patient's intestinal microflora. To our knowledge, this is the first report on the application of the Ds value to the study of intestinal bacteria in faecal samples from UC patients, patients’ consanguineous and non-consanguineous relatives. Future studies should look for bacterial species, which are associated with the aetiology of IBD or otherwise are protective. Likewise, understanding the mechanisms by which intestinal mirobiota contribute to the exacerbation of IBD should reflect significant progress.

## Abbreviations

ANOVA: Analysis of variance; CAI: Clinical activity index; CD: Crohn’s disease; Df: Discriminant function coefficient; DNA: Deoxyribonucleic acid; Ds: Discriminant score; IBD: Inflammatory bowel disease; OTU: Operational-taxonomic-unit; PCR: Polymerase chain reaction; TNF: Tumour necrosis factor; TRF: Terminal restriction fragments; T-RFLP: Terminal restriction fragment length polymorphism.

## Competing interests

The authors declare that they have no competing interest.

## Authors’ contributions

Katsuyuki Fukuda, MD, PhD, was fully involved in the conception, study design, patient management, acquisition and interpretation of the data, statistics, drafting, and preparation of the final manuscript version. Yoshiyuki Fujita, MD contributed to collection of test samples, interpretation of the data and critical review of the final manuscript version. All laboratory assays were commissioned by TechnoSuruga Laboratory Co., Ltd., Shizuoka, Japan and were paid for by the authors’ institute. Both authors read and approved the final version of the manuscript.

## Pre-publication history

The pre-publication history for this paper can be accessed here:

http://www.biomedcentral.com/1471-230X/14/49/prepub
